# 

**Published:** 1899-08

**Authors:** 


### CONTENTS.

COMMUNICATIONS.

Periosteal Caries from Bacterial Origin.... 349
Infectious Ulcerative Stomatitis .... 360
Some Points on the Etiology, Pathology and Treatment of Persistent Pyorrhea .... 368
Epithelial Structures in the Peridental Membrane.... 374

EDITORIAL.

American Dental Association.... 393
National Association of Dental Faculties.... 394
An Important Movement.... 395
The Examiners Meeting.... 395
Johnson's Universal Cyclopedia.... 396

